# **Development of thermo-reporting nanoparticles for accurate sensing of geothermal** reservoir conditions

**DOI:** 10.1038/s41598-020-68122-y

**Published:** 2020-07-10

**Authors:** Bastian Rudolph, Jonathan Berson, Sebastian Held, Fabian Nitschke, Friedemann Wenzel, Thomas Kohl, Thomas Schimmel

**Affiliations:** 1 Institute of Nanotechnology (INT), Material Research Center for Energy Systems (MZE), Institute of Applied Physics (APH), Karlsruhe Institute of Technology, 76131 Karlsruhe, Germany; 2 Institute of Applied Geosciences (AGW), Karlsruhe Institute of Technology, 76131 Karlsruhe, Germany; 3 Geophysical Institute (GPI), Karlsruhe Institute of Technology, 76187 Karlsruhe, Germany

**Keywords:** Nanoscale materials, Nanoparticles, Organic-inorganic nanostructures, Synthesis and processing, Geology, Chemistry, Materials science, Nanoscience and technology

## Abstract

The inaccessibility of geological reservoirs, both for oil and gas production or geothermal usage, makes detection of reservoir properties and conditions a key problem in the field of reservoir engineering, including for the development of geothermal power plants. Herein, an approach is presented for the development of messenger nanoparticles for the determination of reservoir conditions, with a proof of concept example of temperature detection under controlled laboratory conditions. Silica particles are synthesized with a two-layer architecture, an inner enclosed core and an outer porous shell, each doped with a different fluorescent dye to create a dual emission system. Temperature detection happens by a threshold temperature-triggered irreversible release of the outer dye, thus changing the fluorescence signal of the particles. The reported particle system consequently enables a direct, reliable and fast way to determine reservoir temperature. It also displays a sharp threshold for accurate sensing and allows detection at concentration ranges as low as few nanograms of nanoparticles per milliliter.

## Introduction

Determination of various conditions and parameters of geological reservoirs such as temperature, pH or pressure is of utmost importance, both for the assessment of the profitability of underground usage in terms of geothermal power generation or oil and gas production, as well as for the management and maintenance of running facilities^1,2^. However, the inaccessibility of such reservoirs, buried kilometers underground and located in dense lithologies impossible to penetrate by any measurement equipment, make reservoir characterization a complicated task^3,4^. Beyond the immediate vicinity of wellbores, where the parameters are determined by direct downhole measurement techniques^5^, underground reservoirs are mainly explored by injecting solute tracers into the reservoir and retrieving them at an exit point. The most basic tracers, some of them used as early as the beginning of the twentieth century^6^, were utilized mainly for determining flow path and rates^7^. More elaborate chemical tracers, often referred to as ‘smart tracers’, detect reservoir conditions such as temperature, by a chemical transformation that is induced upon experiencing certain condition within the reservoir^8,9^. However, chemical tracers have to be used in large quantities in order to be detectable at the recovery point, as just a low fraction of the injected amount is recovered at the exit point due to interaction with and sorption on the reservoir rock^10–12^. The reservoir fluids are also highly mineralized and may mask the signal of the tracer. Moreover, depending on reaction kinetics, most chemical modifications of molecules do not have a sharp threshold and it may be hard to distinguish between e.g., long exposure of the tracer to a moderate temperature and short exposure to a high temperature. For example, a work by Maier et al.^13^ demonstrates the intricate relationship between the hydrolization of thermo-sensitive tracers at temperatures ranging from 40 to 60 °C and the time of exposure to the relevant temperature. A meaningful interpretation of smart tracer experiments requires a priori knowledge of the reservoir parameters such as flow rate, temperature, pH or length of the flow path that is often lacking. Thus, the results based solely on the assessment of the smart tracer cannot stand on their own^13,14^.

Utilization of nanoparticles in reservoir characterization bears the promise of helping to overcome some of these issues, while offering many additional possibilities for the development of smart and versatile exploration tools. First and foremost, while being small enough to be able to flow through porous rock layers^15^, nanoparticles can still concentrate a substantial amount of molecules, making detection feasible while using much lower tracer amounts^16^. Moreover, multiple chemical markers can be incorporated into the same nanoparticles, enabling the addition of a stable background signal alongside a reporting signal^17–19^. Consequently, each nanoparticle can become a complete system with an inherent reference signal that is compared to a different, stimuli-responsive, reporting function. Thus, the ratio between the reporting and the reference signals helps overcome the need to know the amount of recovered tracer and reduces the dependency on complicated measurement and simulation of reservoir properties.

Fluorescent dyes were selected for the signaling/reference and reporting functions, as they can be detected at low concentrations with a high signal-to-noise ratio and are less susceptible to false detection as a result of signals emanating from other chemicals in the reservoir fluid. Since the combination of a specific range of excitation and emission wavelengths of a given fluorescent dye is rare, the use of fluorescent dyes in geothermal systems forms an almost unique nanoparticle fingerprint. Moreover, fluorescence spectroscopy is fast, cheap and simple, which may prove especially advantageous for routine geothermal well real-time monitoring tasks.

## Results and discussion

**Figure 1:**
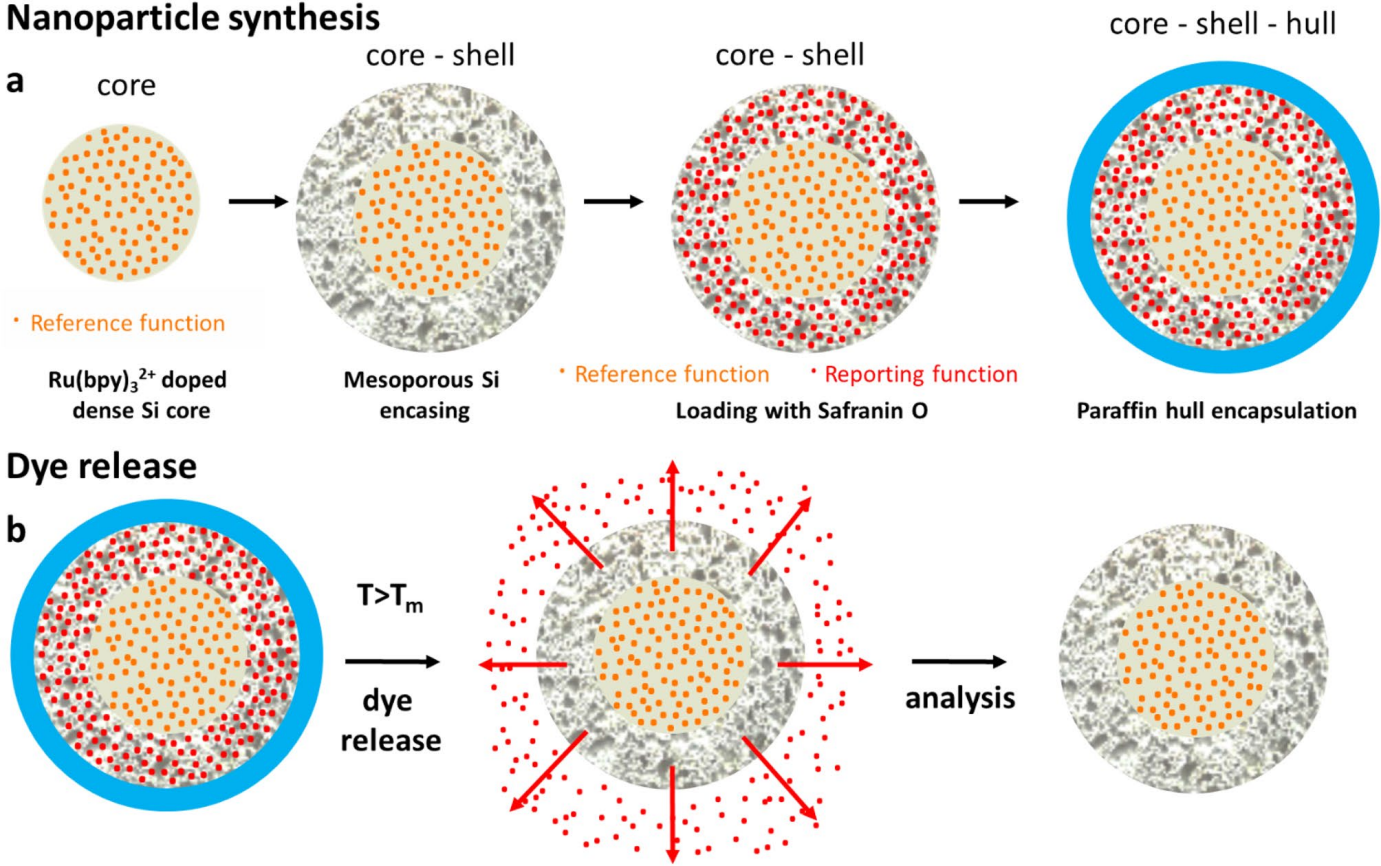
(**a**) A schematic representation of the synthesis stages of the core–shell–hull nanoparticles. (**b**) schematic representation of the dye release from the shell of the nanoparticles upon exceeding the melting point of the paraffin hull, followed by the analysis of particles with a low report-to-reference ratio after the outer dye had been released.

In this report, we introduce a nanoparticle system for the detection and reporting of temperature inside geological reservoirs and test its functionality under laboratory conditions. The nanoparticles are synthesized with a core–shell architecture, encapsulated by a paraffin hull (Fig. 1a). The core is composed of dense silica, in which a fluorescent dye (Tris(bipyridine)ruthenium(II) chloride, Ru(bpy)_3_^2+^, absorption maximum at 452 nm, emission maximum at 612 nm) is embedded. The shell is made of mesoporous silica loaded with a different fluorescent dye (Safranin O, absorption maximum at 530 nm, emission maximum at 585 nm). The Ru(bpy)_3_^2+^ dye in the core plays a dual role—to signal that a certain quantity of nanoparticles has been recovered at the exit point even when the external dye has been released (*signaling function*) and to provide a stable signal that can be compared to the varying signal of the dye in the shell (reference function).

The Safranin O is trapped inside the nanoparticle by a hull made of paraffin molecules with different melting points (mp). Upon exposure of the particles to temperatures above the melting point of the paraffin, the pores in the silica shell are unblocked and the Safranin O that plays the role of the *reporting function* is released (Fig. 1b), changing the ratio between the fluorescence signals of the Safranin O and the Ru(bpy)_3_^2+^.

**Figure 2:**
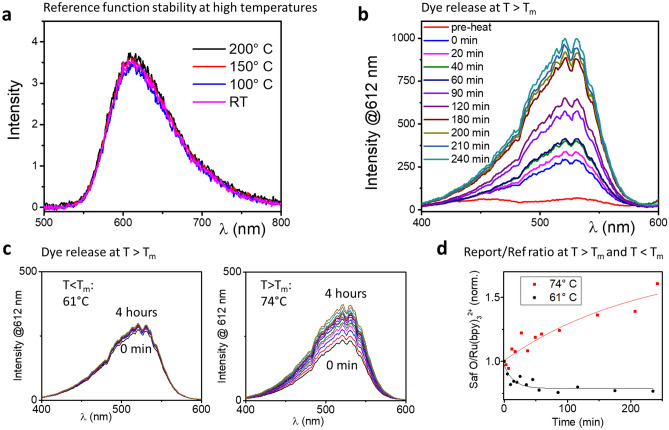
(**a**) Test of the reference signal stability by fluorescence spectroscopy of the signal of the Ru(bpy)_3_^2+^ embedded in the nanoparticle core. Nanoparticle solutions of equal concentrations were exposed over 72 h to conditions of room temperature and atmospheric pressure (purple curve), 100 °C and 1.01 bar (blue curve), 150 °C and 4.76 bar (red curve) and 200 °C and 15.55 bar (black curve). (**b**) In-situ time-resolved fluorescence spectroscopy of a core–shell–tetracosane hull nanoparticle solution, conducted by sweeping the excitation wavelength and measuring the resulting emission at the 612 nm wavelength. The different measurements were taken at the noted times after reaching the solution target temperature of 52 °C with first measurement taken before heating commenced (pre-heat) and the next measurement immediately upon reaching the target temperature (noted as 0 min). (**c**) In-situ time-resolved fluorescence spectroscopy of a core–shell–dotriacontane hull nanoparticle solution, conducted in the same manner as in b. The spectra displayed on the left were obtained over 4 h from a solution heated to 61 °C while the spectra on the right were obtained from a solution heated to 74 °C. (**d**) A graph showing the evolution over time of the signal/ref ratio derived from the fluorescence spectroscopy of the 74 °C (red curve) and 61 °C (black curve) heated solutions displayed on c. The signal/ref ratio was normalized to the ratio at the starting point of the experiment.

The nanoparticle synthesis starts with a modified Stöber synthesis with tetraethyl orthosilicate (TEOS) and (3-Aminopropyl)triethoxysilane (ATPES) as the silica precursors, in the presence of Ru(bpy)_3_^2+^^20^. Since the Ru(bpy)_3_^2+^ is the reference function, which has to be stable throughout the experiment, it is important to ensure that the fluorescent dye does not leak out or bleach over time or when exposed to high temperatures. Figure 2a shows the emission spectrum (excitation wavelength 452 nm) of four 0.1 mg ml^−1^ solutions of Ru(bpy)_3_^2+^ doped silica nanoparticles after 72 h in an autoclave at different conditions ranging from room temperature and atmospheric pressure (purple curve) to 200 °C and a pressure of 15.55 bar (black curve). The fluorescence spectroscopy confirms that the emission intensities of all solutions are similar, regardless of the conditions they had been exposed to, which proves that the silica core effectively encapsulates and protects the signaling/reference function within the inspected temperature and pressure range. Leakage or bleaching would result in an increase or a decrease in the fluorescence signal, respectively. In other words, the fluorescent dye in the core does not leak and is protected from degradation under ambient conditions typical to geothermal reservoirs.

The second step of the synthesis is the addition of cetrimonium bromide (CTAB) and a new dose of TEOS and APTES silica precursors to envelope the core with a silica shell layer. The shell has a template of CTAB micelles entrapped in it, based on a one-pot shell regrowth procedure developed by Ishii et al.^21^. The CTAB micelles are then extracted by reflux in a methanol and hydrochloric acid solution (pH 0.9) to leave pores in the silica shell, which are subsequently loaded with safranin O, before being encapsulated in a paraffin hull^22^. SEM imaging of the obtained particles shows that the synthesis yielded round particles with an average diameter value of 188 ± 59 nm (Supplementary Fig. 1). Reporting tracer particles in this size range can be utilized to evaluate the temperature conditions along flow paths in either fractures (e.g. in crystalline rocks) or the effective pore space in porous rocks (e.g. sandstones)^23^. For instances where the pore throat diameter are smaller, the nanoparticle size can be reduced by changing synthesis parameters.

The Ru(bpy)_3_^2+^ emission of the nanoparticles was detected in clean water solutions at standard measurement conditions (see experimental section) even at concentrations as low as 1.3 ng ml^−1^ with a signal-to-noise ratio of 5.3.

We demonstrate detection of three different threshold temperatures using different paraffin hulls: Tetracosane (CH_3_(CH_2_)_22_CH_3_, mp 49–52 °C), dotriacontane (CH_3_(CH_2_)_30_CH_3_, mp 65–70 °C) and tetratetracontane (CH_3_(CH_2_)_42_CH_3_, mp 85–87 °C). Monitoring of the dye release process by fluorescence spectroscopy was conducted by sweeping the excitation wavelength and measuring the resulting emission at the 612 nm wavelength. While the emission spectra of Ru(bpy)_3_^2+^ and Safranin O largely overlap (Supplementary Fig. 2), the excitation wavelengths are distinctly separated. Both Ru(bpy)_3_^2+^ and Safranin O show significant emission at 612 nm and it is therefore possible to de-convolute the discrete fluorescence contribution of each dye, based on the fluorescence spectra of each dye separately (Supplementary Fig. 3). The ratio of the Safranin O to Ru(bpy)_3_^2+^ fluorescence signals (reporting function to reference function, in short: “*report/ref*”) is calculated for each batch of synthesized particles.

Upon heating the nanoparticles to temperatures above the melting point of the paraffin, the Safranin O stored in the mesoporous silica shell is released into the solution. As a result of the Safranin O molecules no longer concentrated in a confined space, photon absorption efficiency increases and fluorescence quenching decreases, thus leading to an increase in emission and subsequently to an increase in the *report/ref* ratio. Figure 2b shows in-situ fluorescence measurements conducted over 4 h of heating tetracosane-coated nanoparticles to the tetracosane melting point of 52 °C. As Safranin O is released over time, its fluorescence signal increases while that of the embedded leak-proof Ru(bpy)_3_^2+^ stays stable. As a result, the *report/ref* ratio (Ru(bpy)_3_^2+^ to Safranin O fluorescence intensity ratio) increases from an initial ratio of 0.96 to a ratio of 33.5. In Fig. 2c, the results of a similar experiment with dotriacontane-coated nanoparticles are displayed, with a solution that is heated to 61 °C (4 °C below the melting point) on the left and a solution of similar concentration that is heated to 74 °C (4 °C above the melting point) on the right. Over 4 h of heating, the *report/ref* ratio of the 74 °C solution gradually increases from 18.84 to 30.32. In comparison, for the 61 °C, the ratio slightly decreases over the first hour, and stabilizes around values of 15.5. Figure 2d shows a comparison of the evolution over time of the *report/ref* ratio between the 74 °C solution (red curve) and the 61 °C (black curve), normalized to the initial ratio. A summary of the change in the *report/ref* ratio upon heating to above the melting point of the paraffin hulls, for particles with tetracosane, dotriacontane and tetratetracontane hulls, is shown in Table 1. The table also includes the corresponding ratios for unheated particles and particles heated to temperatures slightly below the relevant melting point.

**Table 1:** Ratios of the reporting to the reference fluorescence signal of Ru(bpy)_3_^2+^ core–Safranin O shell–paraffin hull nanoparticles with different paraffin hulls at room temperature, heated slightly below the mp of the paraffin hull and slightly above it.

Type of nanoparticle hull (mp in °C)	Report/ref ratio at room temperature	Report/ref ratio slightly below mp	Report/ref ratio slightly above mp
Tetracosane (49–52)	0.95	4.15 (at 45 °C)	33.5 (at 52 °C)
Dotriacontane (65–70)	18.8	15.5 (at 61 °C)	30.3 (at 74 °C)
Tetratetracontane (85–87)	4.04	5.31 (at 81 °C)	43.3 (at 89 °C)

To ensure that the nanoparticle system is stable and the paraffin hull stays intact under typical geothermal conditions, control experiments were conducted under varying pH values in highly saline solutions. To emulate the salinity and pH levels of typical geothermal fluids^24,25^, dotriacontane-coated nanoparticle were dispersed in 50 g l^−1^ sodium chloride solutions, which were then modified to pH values of 3 and 10 using hydrochloric acid and sodium hydroxide, respectively. The solutions of pH values 3 and 10, as well as a reference sample of pH 7, were kept at room temperature for a week and did not show any sign of dye release. Parallel samples that were heated to 74 °C were activated and released the dye in a similar manner to particle solutions in clean water (Supplementary Fig. 4 and Supplementary Table 1).

**Figure 3:**
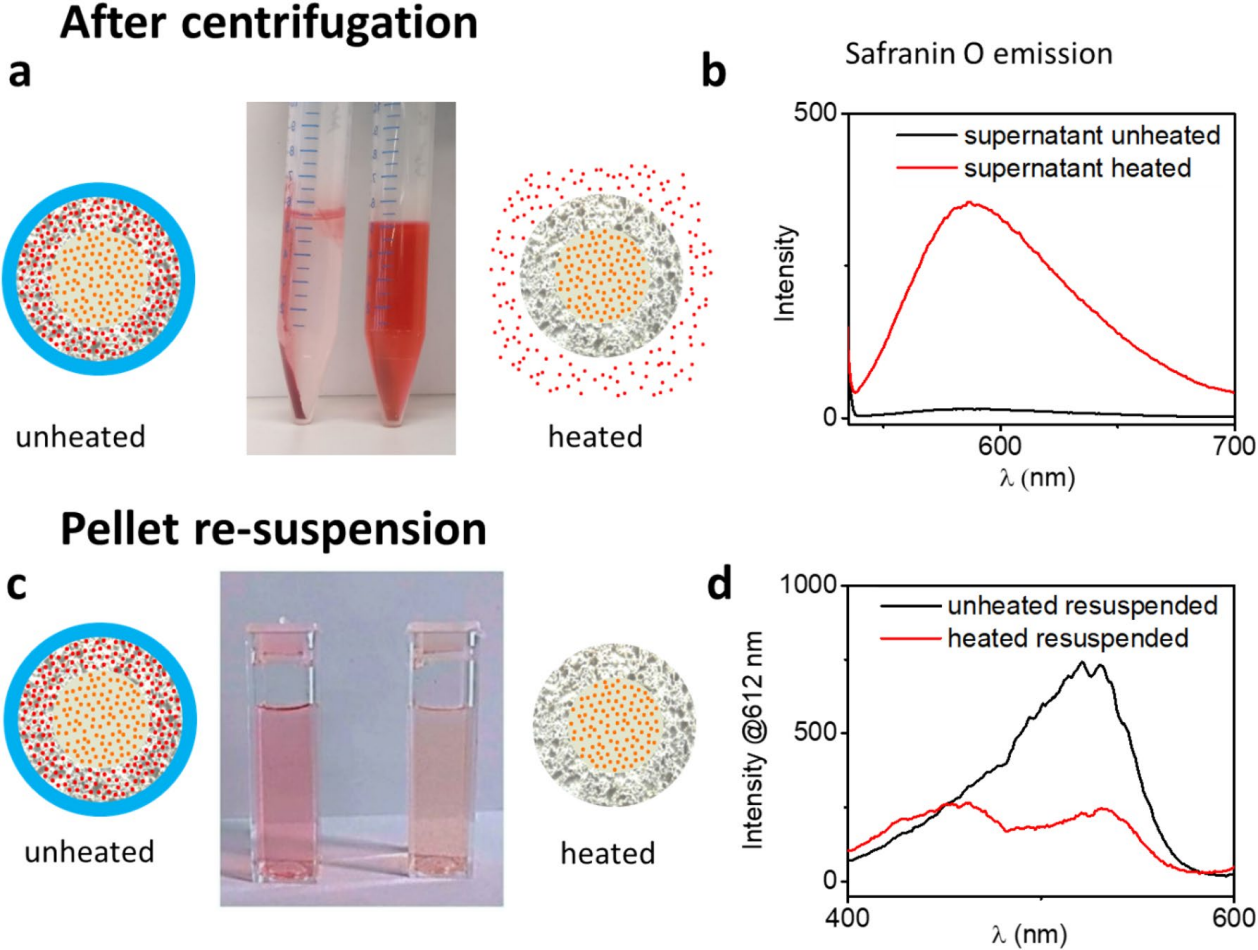
(**a**) A picture of centrifugation tubes containing the pellets and supernatants of solutions of unheated (left) and heated (74 °C, right) solutions of core–shell–hull (dotriacontane) nanoparticles after centrifugation, with the corresponding schematic representations of the state of the nanoparticles. (**b**) The fluorescence emission signal of the supernatant obtained from the centrifugation tubes, upon excitation at 530 nm. (**c**) An image of cuvettes containing solutions of the re-suspended nanoparticles taken from the pellets in the centrifugation tubes. (**d**) Fluorescence spectroscopy of the solutions displayed in (**c**), conducted by sweeping the excitation wavelength and measuring the resulting emission at the 612 nm wavelength.

As a further demonstration of the selective dye release process from the nanoparticle shell upon heating, Fig. 3a shows two centrifugation tubes containing dotriacontane-coated nanoparticle solutions of identical concentrations (2 mg nanoparticles per ml) , after centrifugation. The tube on the left was held at room temperature while the tube on the right was heated to 74 °C. As is even evident to the naked eye, fluorescence spectroscopy of the supernatant after centrifugation (excitation wavelength 530 nm) shows that a significantly larger amount of safranin O was released in the heated solution while only traces were detected in the room temperature solution (Fig. 3b). No Ru(bpy)_3_^2+^ was detected in either of the solutions.

While the results demonstrating that dye release indeed takes place upon heating to temperatures above the melting point of the paraffin hull are important, detection onsite after reservoir percolation would not be dependent on identification of the dye release itself but rather on identification of the recovered nanoparticles. In the same experiment shown in Fig. 3a, the nanoparticle pellets were dried and re-suspended to make new solutions of similar concentrations. The room temperature nanoparticles had a pink hue, typical of the Safranin O dye, and displayed a *report/ref* ratio of 19.01 whereas the heated nanoparticles had an orange tone, typical of the Ru(bpy)_3_^2+^ dye, and displayed a *report/ref* ratio of 0.99 (Fig. 3 c, d).

## Conclusions

To summarize, we present a facile approach to advance geological reservoir characterization, which is based on the combination a number of principles: the synthesized nanotracers have a signalling function for detection upon recovery. The dye-doped core also functions as robust reference function, leak-proof and stable, making measurement analysis straight-forward and independent of the need for additional data or simulations. The presented nanotracers encompass a stimuli responsive system, triggered by a sharp threshold and inducing an irreversible change in the reporting function. Fluorescence signalling is utilized to ensure a distinctive fingerprint of the nanotracers for detection even against complex background matrices. In addition, fluorescence signalling enables high detection sensitivity and fast and simple operation, suitable for constant monitoring of geothermal fluids at the exit point. While this work was conducted with a basic fluorescence spectroscopy setting, detection sensitivity and accuracy may be enhanced even further by employing more sophisticated equipment or advanced techniques such as laser induced fluorescence^26^.

This report is based on a proof-of-principle example of temperature detection with a certain architecture of silica core–shell nanoparticles. Looking towards future application, the diverse synthetic routes available may pave the way towards development of various nanotracers, specifically adapted to test and withstand the conditions of particular reservoirs. These would be distinguished from one another by different combinations of fluorescent reference and reporting functions for different melting points or triggers based on other properties, altered sizes for different flow paths inside the reservoir or different stimuli responsive mechanisms for detection of various reservoir parameters.

We believe that the reported approach could not only expand the extent and accuracy of information acquired in geothermal exploration but also prove to be a cost-effective one. The synthesis is completed within 3 days and is based on affordable chemicals, the most expensive of which are the dyes, which are also used in any traditional tracer test. In addition, when comparing nanoparticle to chemical tracer tests, we consider that the high detection sensitivity of particles, which confine many dye molecules in a small space, would significantly reduce the total amount of dye that would be required for a successful test.

When considering possible environmental implications of the reporting nanotracers, lesser dye use would also have a smaller environmental impact and potential hazard. Silica nanoparticles are not considered a major health risk and are actually used in the development of various medical applications^27^. It should also be noted that in any case geothermal reservoirs are normally highly mineralized, often radioactive and the environment is not meant to be exposed to these fluids, nor are geothermal fluids supposed to mix with sources of potable water.

## Methods

### Core–shell–hull nanoparticles synthesis

The silica core synthesis starts with a mixture of 9.5 ml H_2_O, 1.45 ml ethylenglycol (Sigma Aldrich, 99.8%,0.42 ml 28–30% ammonium hydroxide solution (Merck), stirred and heated to 60 °C. 25 mg of Tris(2,2-bipyridyl)dichlororuthenium(II) hexahydrate (Ru(bpy)_3_^2+^) is added, and then 0.33 ml of Tetraethyl orthosilicate (TEOS, Aldrich, 99.0%), which is gradually added dropwise over one minute. 30 min later, 60 µl of (3-Aminopropyl)triethoxysilane (ATPES, Aldrich, 99%) are added. After 4 h of continued stirring at 60 °C, Ru(bpy)_3_^2+^-doped silica nanopartiocles (SiNPs) are formed.

The synthesis of the silica shell, which contains the reporting function, proceeds in the same reaction pot by the addition 60 mg of cetrimonium bromide (CTAB, Merck, 97.0%) and 30 min later of a mixture of 0.33 ml TEOS and 60 µl APTES. After a further 90 min, the inner Ru(bpy)_3_^2+^-doped SiNPs are coated with an external layer of silica with CTAB micelles entrapped in it. The solution is then collected, centrifuged at 6,000 rpm, and undergoes 3 wash-centrifugation cycles with H_2_O, ethanol and methanol as solvents. The particles are then resuspended in methanol and the CTAB micelles are extracted by refluxing for 16 h in a mixture of 120 ml methanol (VWR Chemicals, 99.9%), 2.48 ml H_2_O and 1.24 ml hydrochloric acid (HCl, Fluka 36.5–38%) under stirring and heating to 70 °C. The particles are then collected by centrifugation at 6,000 rpm and washed by methanol and ethanol. The particles are then vacuum dried for at least 3 h and weighed. They are stored in a water free environment (desiccator purged with dry nitrogen or argon or in a glovebox with H_2_O levels lower than 50 ppb), where 17 mg safranin O (Acros Organics, 95%) is added to 50 mg of NPs. After 2 h, 2.5 ml of dry acetonitrile (Merck, 99.5%) is added, the solution is sealed to prevent adsorption of water from the air and is stirred for at least 12 h. 0.375 ml of n-Octadecyltrimethoxysilane (ABCR GmbH, 95%) is added to enable the adsorption of the paraffin layer later on. The solution is further stirred for at least 12 h, then the NPs are collected by centrifugation and are washed with acetonitrile (Merck, 99.5%), following which they are vacuum dried. When the NPs are dry they are washed and centrifuged in hexane (Carl Roth, 99%), then dispersed in 40 ml hexane. 375 mg of paraffin is added to the mixture (all 3 paraffins are manufactured by Aldrich, Tetracosane 99%, Dotriacontane 97%, tetratetracontane 99%) in accordance with the temperature threshold that is required (see Table 1). The mixture is sonicated for 15 min and stirred for 15 min, following which the paraffin-coated NPs are collected by centrifugation and dried in vacuum. When the NPs are dry they are re-suspended in water with sodium dodecyl sulfate (SDS, Sigma-Aldrich 99.0%) and undergoe centrifugation—H_2_O wash cycles until the residual safranin O is washed away and the supernatant is clear. The particles are then ready for use.

### Nanoparticles dispersion

The nanoparticles may be difficult to dissolve along the steps of the above-described multiple-step synthesis, especially after they were vacuum dried or centrifuged. To re-suspend the particles, the reaction vessels or centrifugation tubes were vortexed and sonicated. When needed, a sonotrode (IUP200St, Hielscher Ultrasonics) was applied as well. This process was regulated by a temperature sensor immersed in the solution to make sure the solution does not exceed the mp of the paraffin during the sonication process. Likewise, longer sonications in sonication baths were performed while maintaining the bath water at room temperature using a circulation thermostat.

### Fluorescence spectroscopy

Fluorescence spectroscopy was conducted using a Cary Eclipse Fluorescence Spectrophotometer (Agilent Technologies, USA). In-situ heating was conducted by warming the sample holder with a circulation of heating liquid and immersing a temperature probe in the nanoparticle solution. All measurements were performed in a 1 cm × 1 cm quartz fluorescence cuvettes.

## Supplementary information

Supplementary file1 (DOCX 771 kb)
