# Supplementary material for: Development of thermo-reporting nanoparticles for accurate sensing of geothermal reservoir conditions
Source: Sci Rep. 2020 Jul 10;10:11422. doi: 10.1038/s41598-020-68122-y (PMC7351951; doi:10.1038/s41598-020-68122-y)
Supplement: Supplementary file 1 — Supplementary file1 (DOCX 771 kb) [file 41598_2020_68122_MOESM1_ESM.docx]

Supplementary Information

Development of thermo-reporting nanoparticles for accurate sensing of geothermal reservoir conditions

Bastian Rudolph^1^, Jonathan Berson^1,*^, Sebastian Held^2^, Fabian Nitschke^2^, Friedemann Wenzel^3^, Thomas Kohl^2^ and Thomas Schimmel^1^


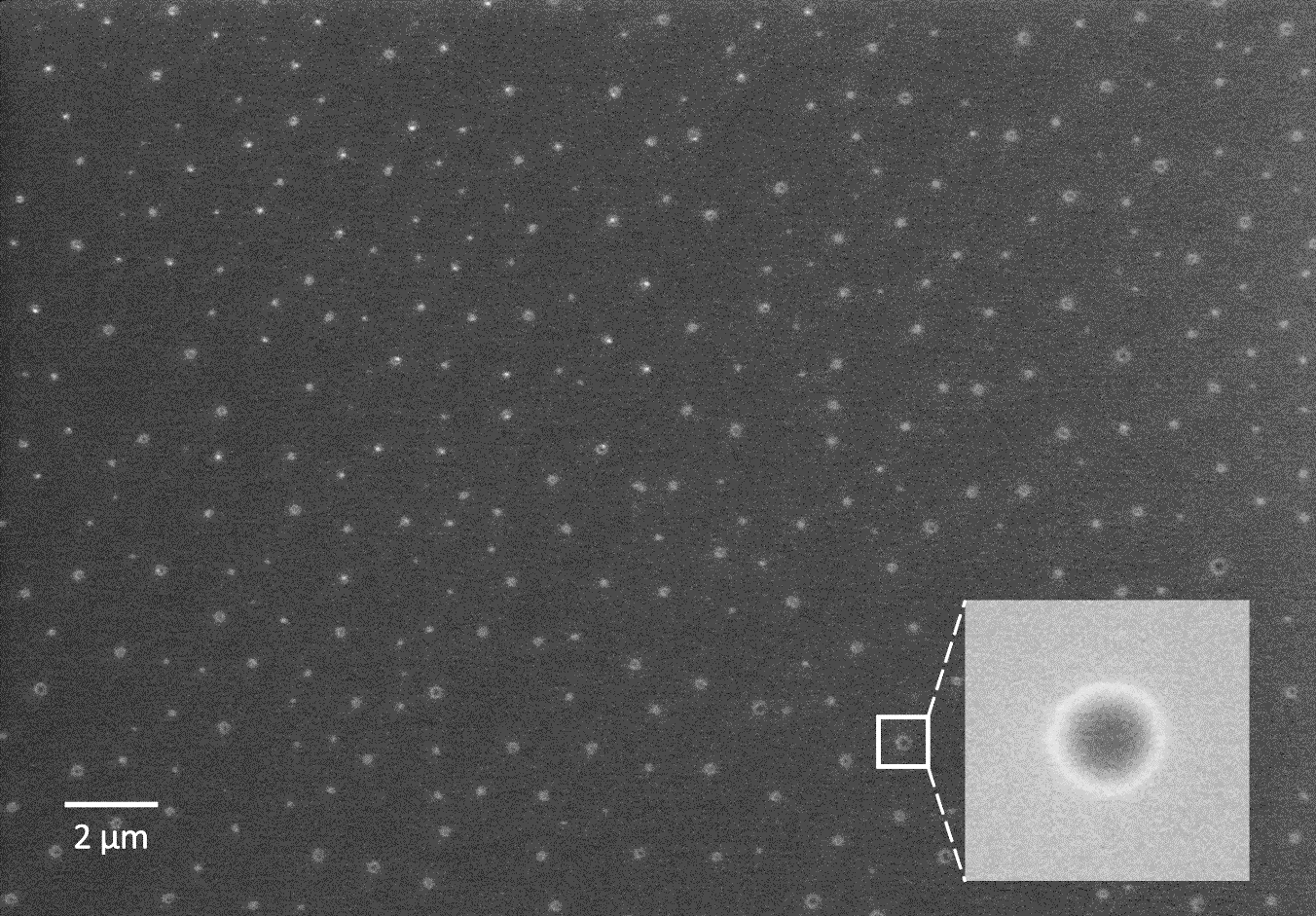


**Fig. S1**. SEM image of the core – shell – hull (dotriacontane) messenger nanoparticles.

**Fig. S2**. The overlapping fluorescence emissions of Safranin O (upon excitation at a wavelength of 530 nm, black curve) and Ru(bpy)_3_^2+^ (upon excitation at a wavelength of 452 nm, red curve).

**Fig. S3**. Fluorescence spectroscopy of Ru(bpy)_3_^2+^ (black curve) and safranin O (red curve) dye solutions, conducted by sweeping the excitation wavelength and measuring the resulting emission at the 612 nm wavelength.


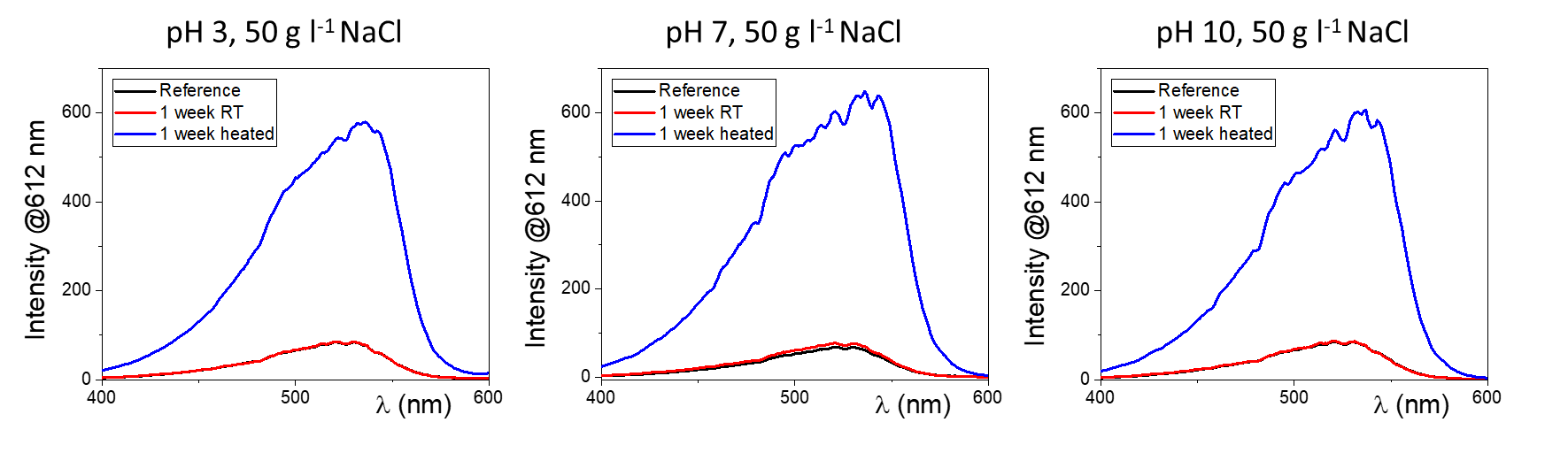


**Fig. S4**. Test of the stability and operation of the reporting nanoparticles at high salinity and different pH level. Solutions of 50 g l^-1^ NaCl and core - shell – dotriacontane particles were prepared and modified to pH values of 3 (left), 7 (middle) and 10 (right). The fluorescence signal of the different solutions was measured once upon preparation (black curves) and then after a week (red curves), as well as after heating to 74 °C (blue curves). The measurements were conducted by sweeping the excitation wavelength and measuring the resulting emission at the 612 nm wavelength.

**Table S1.** Ratios of the reporting to the reference fluorescence signal of Ru(bpy)_3_^2+^ core - Safranin O shell – dotriacontane hull nanoparticles in 50 gr/l NaCl solutions with different pH levels. The ratios were measured upon preparation, after a week and after heating to 74 °C.

| pH level | report/ref ratio  after preparation | report/ref ratio  after a week | report/ref ratio  after heating |
| --- | --- | --- | --- |
| 3 | 10.36 | 9.83 | 20.82 |
| 7 | 9.82 | 10.24 | 21.15 |
| 10 | 9.32 | 9.27 | 20.22 |
